# Electrospinning of Aqueous Solutions of Atactic Poly(*N*-isopropylacrylamide) with Physical Gelation

**DOI:** 10.3390/gels8110716

**Published:** 2022-11-05

**Authors:** Ya-Chen Chuang, Yu-Chia Chang, Meng-Tzu Tsai, Ting-Wei Yang, Meng-Tse Huang, Shao-Hua Wu, Chi Wang

**Affiliations:** Department of Chemical Engineering, National Cheng Kung University, Tainan 70101, Taiwan

**Keywords:** physical gelation, phase separation, phase diagram, electrospinning, nanofibers

## Abstract

The phase diagram of a given polymer solution is used to determine the solution’s electrospinnability. We constructed a phase diagram of an aqueous solution of atactic poly(*N*-isopropylacrylamide) (a-PNIPAM) based on turbidity measurements and the rheological properties derived from linear viscoelasticity. Several important transition temperatures were obtained and discussed, including the onset temperature for concentration fluctuations *T*_1_, gel temperature *T*_gel_, and binodal temperature *T*_b_. On heating from 15 °C, the one-phase a-PNIPAM solution underwent pronounced concentration fluctuations at temperatures above *T*_1_. At higher temperatures, the thermal concentration fluctuations subsequently triggered the physical gelation process to develop a macroscopic-scale gel network at *T*_gel_ before the phase separation at *T*_b_. Thus, the temperature sequence for the transition is: *T*_1_ < *T*_gel_ < *T*_b_~31 °C for a given a-PNIPAM aqueous solution. Based on the phase diagram, a low-temperature electrospinning process was designed to successfully obtain uniform a-PNIPAM nanofibers by controlling the solution temperature below *T*_1_. In addition, the electrospinning of an a-PNIPAM hydrogel at *T*_gel_ < *T* < *T*_b_ was found to be feasible considering that the elastic modulus of the gel was shown to be very low (ca. 10–20 Pa); however, at the jet end, jet whipping was not seen, though the spitting out of the internal structures was observed with high-speed video. In this case, not only dried nanofibers but also some by-products were produced. At *T* > *T*_b_, electrospinning became problematic for the phase-separated gel because the enhanced gel elasticity dramatically resisted the stretching forces induced by the electric field.

## 1. Introduction

Electrospinning is a convenient process for producing polymeric fibers with submicron diameters. The nanofibers of different polymers can be readily obtained for various applications by using this technique [1,2,3,4]. In general, semi-dilute solutions with a sufficient entanglement density are required to yield bead-free fibers [5,6]. However, for some polymer solutions, such as aqueous solutions of poly(*N*-isopropylacrylamide) (PNIPAM), the successful production of bead-free fibers is difficult to achieve [7,8]. This processing difficulty is attributed to the complex phase behavior of PNIPAM/H_2_O at an ambient temperature of 20–33 °C. A previous study on the electrospinning of PNIPAM aqueous solutions at ambient temperature showed that broken fibers of short lengths were observed on the collector [7]. Moreover, the cross-sections of as-spun PNIPAM fibers appear to be dog-bone-like and/or ribbon-like rather than the desired circular shape. The formation of a ribbon-like cross-section is often attributed to the intermediate stage of skin/core jet morphology with a solid-like skin that encloses the fluid core during electrospinning [9]. Hence, this proposed mechanism is widely applicable to polymer solutions with volatile solvents, such as chloroform and THF, because of rapid solvent evaporation at the jet/air interface. However, further investigation is needed for cases when non-volatile solvents are used, such as in the case of the present PNIPAM aqueous solutions.

When electrospinning is performed at temperatures that are close to the phase separation temperature (or gel temperature), non-uniformity in polymer concentration along the spinline is expected, thus causing difficulties in electrospinning. Once the flow-induced concentration fluctuations are further enhanced [10], the uniform stretching of the electrospinning jet becomes infeasible. Then, jet portions containing a polymer-lean phase with a low entanglement density can be easily fractured by the electrical field-induced stretching forces, thereby yielding short fibers on the grounded collector after solvent evaporation. Based on these arguments, investigating the rheological properties and the related phase diagram of PNIPAM/H_2_O solutions is required before electrospinning can be used to successfully produce long fibers.

PNIPAM is a thermoresponsive polymer that has attracted increasing attention in practical applications for smart materials and biomaterials [11,12]. PNIPAM is water-soluble at low temperatures but becomes water-insoluble at elevated temperatures, thus exhibiting the phase behavior of a lower critical solution temperature (LCST). The mechanism of the phase separation of PNIPAM/H_2_O has been extensively investigated in the past five decades [13,14,15,16,17,18,19,20,21,22,23,24,25]. Based on a review paper [14], the LCST of PNIPAM is ~31 °C with a critical concentration of 16 wt%. At temperatures lower than LCST, PNIPAM dissolves in water and possesses an expanded chain conformation because of the formation of the “hydrogen bond bridge” developed by the bound water molecules around hydrophobic isopropyl moieties. The hydration of hydrophobic moieties has been often reported for amphiphilic polymers, such as proteins. Then, once the temperature is above the LCST, the free energy change of mixing becomes positive, thus giving rise to solution phase separation.

The formation of the hydrogen bond bridge (or water shell) plays a key role in the LCST behavior of PNIPAM aqueous solutions. Okada and Tanaka proposed a “pearl-necklace” model of PNIPAM chains to successfully predict the square-shaped phase boundary [16]. In the pearl-necklace chain model, the backbone of a PNIPAM chain is composed of two different segments: one is hydrophilic, sheathed with bound water by the fully hydrated amide groups, and the second is in the absence of the bound-water shell; the former exhibits an extended chain conformation (the necklace), while the latter exhibits a random-coiled chain conformation (the pearl). Therefore, understanding the interplay of bound water and free water molecules along long PNIPAM chains is crucial to further grasp the LCST behavior of PNIPAM. The results of the dielectric relaxation spectrum described by Ono and Shikata [26] indicated that the “hydrogen bond bridge” remains dynamically stable up to a temperature of 30 °C, thereby suggesting that the “necklace” portion of the PNIPAM chain is well-protected and is unaffected at *T* ≤ 30 °C. This finding seems to be consistent with the *θ* temperature at ~30.59 °C measured by Kubota et al. [27]. In the past five decades, various advanced techniques have been used to derive the phase transition temperature of PNIPAM/H_2_O solutions [28,29,30,31,32]. Interested readers may refer to a recent review article that provides an in-depth discussion of the phase diagram [14].

Despite extensive studies, the phase separation behavior of PNIPAM/H_2_O is still elusive [14]. The formation of physical gels is known [8,14,33,34], but the gel structure and gelation mechanism are not yet fully studied. Rheometry has been validated to feasibly trace the subtle structure variation of polymer solutions because physical gelation occurs [35,36]. During the evolution of gelation, the enhanced elasticity of a polymer solution can be detected by rheological measurements [37]. Meanwhile, in our recent work, we unambiguously validated the development of thermoreversible physical gels of a-PNIPAM/H_2_O solutions at elevated temperatures lower than *T*_b_ using atactic PNIPAM (a-PNIPAM) with a high molecular weight of 6.58 × 10^5^ g/mol [38]; the gel point was readily determined by the Winter-Chambon criterion with the rheological data obtained from an isothermal frequency sweep test. It should also be noted that gel formation occurs through a liquid-solid transition and that the formation of a “macroscopic physical gel” tends to significantly deteriorate solution spinnability.

In general, a one-phase homogeneous solution with uniform properties is more desirable than a phase-separated solution for electrospinning to obtain uniform nanofibers. However, it should be noted that “flow-induced” phase separation in an electrospinning jet is also likely to occur for a one-phase solution, provided that the stretching rate of the jet is higher than the intrinsic relaxation rate of the polymer solution [10]. A new fiber formation mechanism was recently proposed based on flow-induced phase separation in the spinline [39]. During electrospinning, a polymer solution is delivered at a given flow rate into a capillary (i.e., needle) connected to a high-voltage source. At a critical voltage such that the induced electric stress is sufficiently high to outweigh surface tension, the liquid meniscus at the capillary end forms a conical shape known as a Taylor cone. Moreover, a charged jet is ejected from the cone apex to develop a tapered straight jet. The straight jet (several millimeters long) is affected by the jet-whipping process at its end because of “bending instability” [4,40]. During jet whipping, most of the solvent is subsequently removed, thus leaving charged solid fibers to be collected by a grounded collector. The governing variables to determine the morphology and diameter of electrospun fibers can be generally classified into two groups [1,2,3,6,41], namely, solution properties (e.g., viscosity, conductivity, and surface tension) and processing parameters (e.g., applied voltage, tip-to-collector distance, and solution flow rate).

Previous studies focused on the direct relationship between the governing variables and fiber diameter, but the phase behavior of electrospinning solutions remains largely unexplored. Therefore, this study was mainly aimed to construct the phase diagram of a-PNIPAM aqueous solutions based on rheological properties and cloud points. We succeeded in preparing round a-PNIPAM fibers from aqueous solutions by carefully selecting the appropriate temperature for electrospinning.

## 2. Results and Discussion

In general, one-phase solutions with sufficient entanglement densities are required to obtain a stable cone-jet electrospinning mode for a continuous electrospinning process to obtain uniform nanofibers. Thus, both a phase diagram and information on the entanglement concentration (*ϕ*_e_) must be obtained for a given polymer/solvent pair before electrospinning. In this regard, this paper is organized in the following order. First, we determine the binodal temperature from turbidity measurements, and then we use zero-shear viscosity measurements to derive the *ϕ*_e_. Second, we discuss the rheological data of *G′*(*ω*) and *G″*(*ω*) based on the isothermal frequency sweep to determine the gel point (*T*_gel_) based on the Winter-Chambon criterion. Then, based on our rheological results, a mechanism of physical gelation is proposed. Afterward, we construct a phase diagram for the present system, thus serving as the guideline for the subsequently discussed electrospinning. Finally, the effects of solution status (by varying the solution temperatures) on the electrospinning process were investigated and are discussed.

### 2.1. Determination of Binodal Temperature and Entanglement Concentration

**Figure 1:**
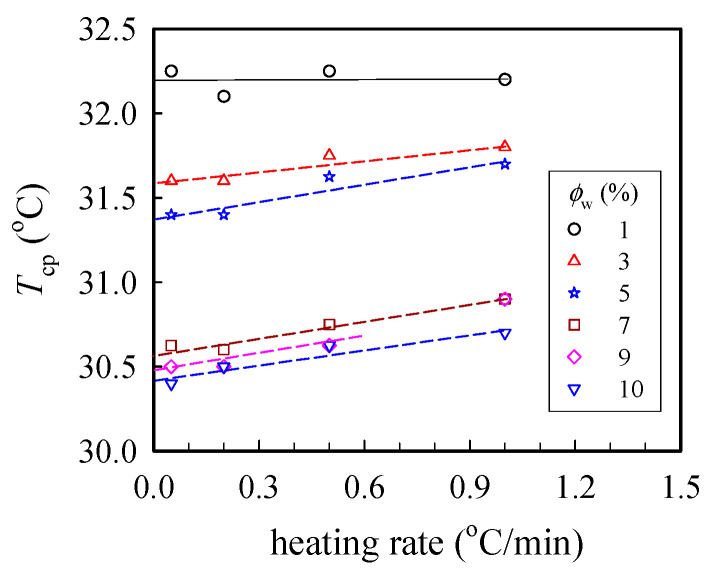
Plots of the cloud point *T*_cp_ at different heating rates for a-PNIPAM/H_2_O of different concentrations *ϕ*_w_. The binodal temperature was derived from the extrapolated temperature at the zero heating rate.

Figure 1 shows the cloud point (*T*_cp_) measured at different heating rates, from which the binodal temperature of the given solution was derived from the *y*-axis intercept via linear extrapolation to the zero heating rate. Herein, *T*_b_ was found to decrease with increasing polymer concentration (*ϕ*_w_); the derived values of *T*_b_ were 32.2 and 30.4 °C for the 1 wt% and 10 wt% solutions, respectively. Thus, based on the turbidity measurements, the transparent sample was validated at *T* < 30 °C regardless of the *ϕ*_w_ studied in this work.

**Figure 2:**
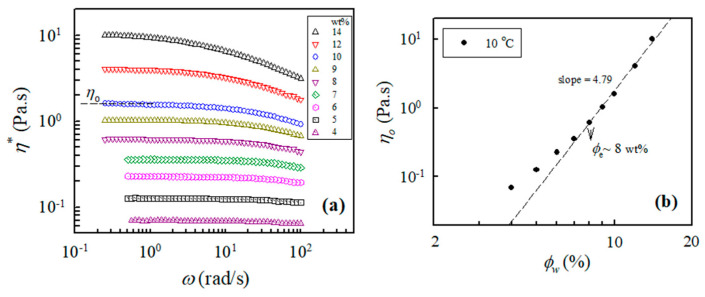
(**a**) Complex viscosity of a-PNIPAM/H_2_O of different concentrations measured at 10 °C; (**b**) log-log plot of *η*_0_ vs. *ϕ*_w_ used to determine the entanglement concentration *ϕ*_e_.

In addition, the measured values of the complex viscosity of the a-PNIPAM aqueous solutions at 10 °C and different frequencies are presented in Figure 2a, from which the zero-shear viscosity *η*_0_ was derived from the constant η^*^ in the low-frequency region, that is, the Newtonian flow region. It should be noted that all the studied solutions (4–14 wt%) at 10 °C were in the one-phase solution state (discussed later). Figure 2b shows the log–log plots of *η*_0_ vs. the polymer concentration to determine the *ϕ*_e_ of the studied a-PNIPAM solutions; the slope increased at low *ϕ*_w_ and finally reached a constant slope of 4.79, thus suggesting the entrance of an entangled solution region. In this manner, *ϕ*_e_ was derived from the incipient concentration at 8 wt%, above which the relationship of *η*_0_ ∝ *ϕ*_w_^4.79^ was maintained. The derived exponent was near the theoretical value of 4.7 for entangled polymers in the *θ* solvent. Our results showed that *ϕ*_e_ was about 8 wt%, which was independent of the solution temperature provided that the a-PNIPAM aqueous solution was in the one-phase state [38].

**Figure 3:**
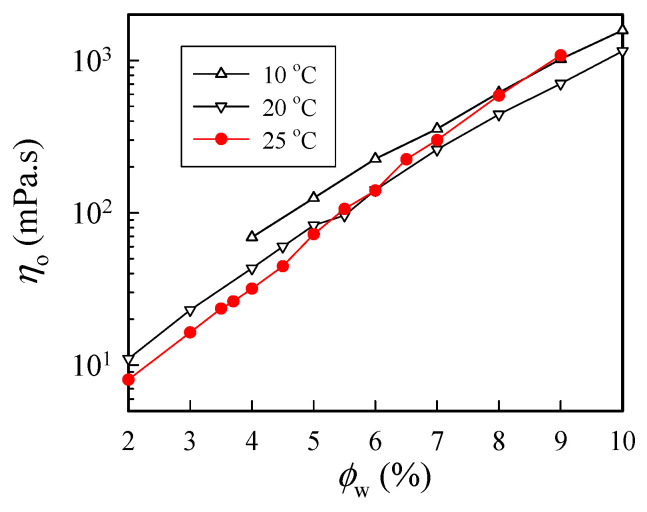
Concentration dependence of zero-shear viscosity of a-PNIPAM/H_2_O measured at different temperatures.

Figure 3 shows the concentration dependence of *η*_0_ measured at three selected temperatures of 10 °C, 20 °C, and 25 °C. As expected, *η*_0_ increased with increasing polymer concentration at a given temperature, and *η*_0_ was larger at 10 °C than that at 20 °C. However, at 25 °C, an anomalous increase in solution viscosity was seen for solutions with a concentration higher than 6 wt%. The solution viscosity of the 9 wt% solutions at 25 °C was even larger than that at 10 °C. Thus, these results indicate that chain structures at 25 °C in the a-PNIPAM/H_2_O solution may have been changed to alter its rheological properties. The anomalous feature arose because of the physical gelation caused by the enhanced chain associations, as *ϕ*_w_ was high.

### 2.2. Determination of Gel Point (T_gel_)

**Figure 4:**
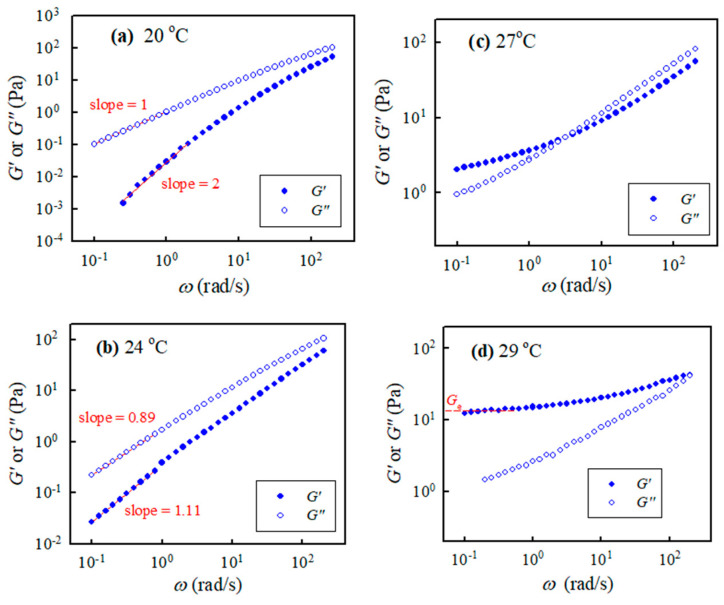
Storage modulus and loss modulus of the 10 wt% a-PNIPAM/H_2_O vs. applied frequencies at different temperatures of (**a**) 20, (**b**) 24, (**c**) 27 and (**d**) 29 °C.

Isothermal frequency sweep tests for selected a-PNIPAM aqueous solutions were carried out at different temperatures with a small temperature increment. Figure 4 shows the *G″*(*ω*) and *G′*(*ω*) curves for the 10 wt% solution at 20 °C, 24 °C, 27 °C, and 29 °C. The a-PNIPAM/H_2_O solutions were homogeneous at 20 °C since terminal flow was reached in the low *ω* region, where *G′* ∝ *ω*^2.0^ and *G″*∝ *ω*^1.0^ were verified. At a higher temperature of 24 °C, the exponent for the *G′*(*ω*) curve in the low *ω* region was reduced to 1.1; the exponent was further reduced to close to zero at 27 °C, thus exhibiting a modulus plateau at *ω* ~0.1 rad/s. The frequency-independent region of the *G′* plateau was extended to *ω* = 1.0 rad/s at 29 °C; the equilibrium plateau modulus *G*_e_ was estimated to be about 13 Pa. More importantly, four-order magnitude increases in *G′* were evident in the low *ω* region, as the temperature was changed from 20 °C to 29 °C (<*T*_b_ = 30.4 °C). In contrast, the *G″*(*ω*) curve merely showed a moderate increase in the low *ω* region. The presence of *G*_e_ at 29 °C indicated the formation of a macroscopic gel of the a-PNIPAM/H_2_O mixture at this elevated temperature (e.g., 29 °C). Hence, significant variations of chain structures occurred in the transparent a-PNIPAM sample of 10 wt% in the small temperature interval of 20–29 °C, as revealed by the dramatic changes in the rheological properties. To derive the gel temperature (*T*_gel_) at which the incipient macroscopic gel was developed, a rigorous approach was required.

**Figure 5:**
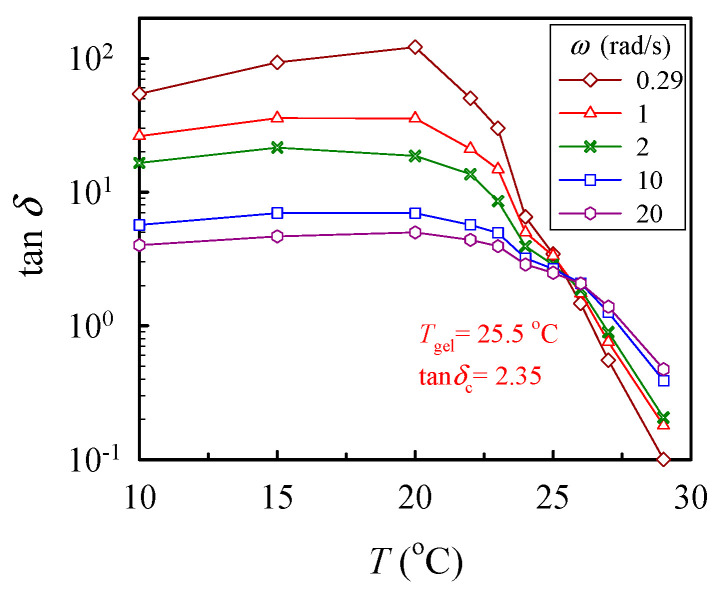
Plot of tan *δ* vs. temperature for the 10 wt% solution measured at different frequencies *ω* to determine the crossover point according to the Winter-Chambon criterion (Equation (2)).

At the gel point (GP), the dynamic modulus of *G′* and *G″* exhibit a power law frequency dependence [36,42]:

(1) $$G^{\prime }\left(\omega \right)\propto G^{\prime \prime }\left(\omega \right)\propto \omega^{n}\;\mathrm{for}\;0<\omega <1/\lambda_{0}$$

and a loss tangent that is independent of frequency,

(2) $$\tan\delta_{c}=\tan\left(n\pi /2\right)$$

where *n* is the critical relaxation exponent and 1/*λ*_0_ denotes the crossover frequency to some faster dynamics (e.g., entanglement region and glass transition) [43]. With a series of frequency sweeps, the Winter-Chambon criterion defined by Equations (1) and (2) was unambiguously applied to identify the GP, at which the macroscopic network was incipiently developed. Based on Equation (2), the plots of tan *δ* (= *G″*/*G′*) as a function of temperature, with the frequency as a parameter, are shown in Figure 5, in which the crossover of the curves marks the gel point. Thus, the 10 wt% solution reached its gel point at 25.5 °C with a corresponding tan *δ*_c_ of 2.35, thus giving rise to an *n* value of 0.76. Similar analyses have been carried out to determine the corresponding values of *n* and *T*_gel_ for other a-PNIPAM solutions of different concentrations [38]. Our work showed that the derived values of *n* were independent of the *ϕ*_w_, while *T*_gel_ decreased with increasing *ϕ*_w_, being 29.5 °C and 25.0 °C for the 5 wt% and 12 wt% solutions, respectively.

**Figure 6:**
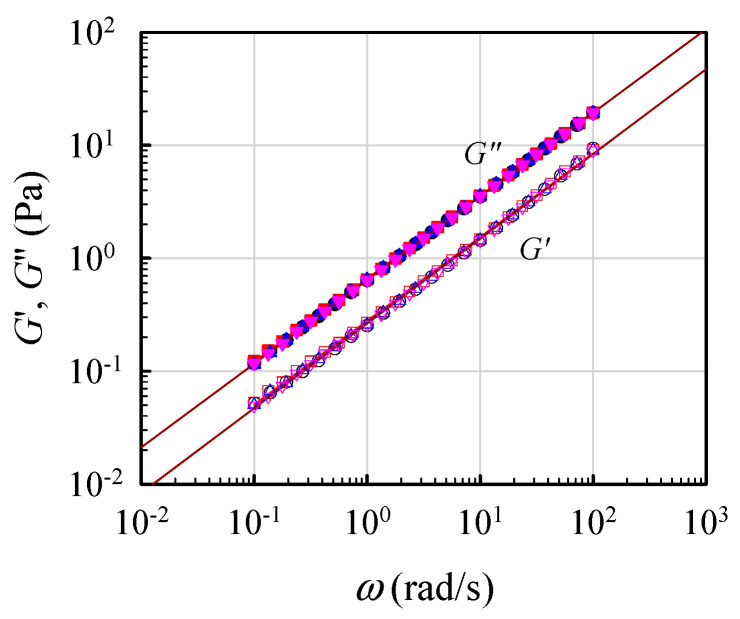
Parallel lines of log G′ and log G″ vs. log *ω* to validate the formation of a critical gel at *T*_gel_ for the 7 wt% solution, according to Equation (1).

To validate the critical gel behavior, frequency sweep tests for the 7 wt% solutions were repeatedly performed at its derived *T*_gel_ for a time period of 12 h. Figure 6 shows the obtained results for four sets of data on *G′*(*ω*) and *G″*(*ω*) measured at 0.5, 4, 10, and 12 h after *T*_gel_ was attained. The superimposed data reveal that the critical gel behavior was unchanged, thus re-confirming that the *G′*(*ω*) and *G″*(*ω*) curves followed Equation (1) with an *n* value of about 0.75. Thus, the critical gel behavior was maintained over the long period of at least 12 h.

As shown above, the physical gelation of a-PNIPAM/H_2_O was found to involve interchain associations used to enhance the elastic response of *G′*(*ω*). More importantly, the derived *T*_gel_ was lower than the *T*_b_ of the corresponding solutions of 5–12 wt%; similar gelation behavior was reported in a binary mixture of isotactic-rich PNIPAM and H_2_O [34] using the ball-dropping method.

An a-PNIPAM hydrogel can be well-described by the Winter–Chambon criterion, thus suggesting that self-similar structures of branched chains are progressively developed before the GP and eventually form a macroscopic 3D network at the GP. Based on percolation theory, in the close vicinity of the GP, the zero-shear viscosity *η*_0_ of the sol in the pregel regime can be described by a scaling law [36]: $\eta_{0}\sim {\left|T-T_{\mathrm{gel}}\right|}^{-s}$, with *s* being a positive exponent. Thus, interchain associations may enhance the *η*_0_ of an a-PNIPAM pregel solution at an elevated temperature before *T*_gel_. In other words, there is a critical temperature, above which profound interchain association occurs and leads to the development of highly branched chains, thereby altering the viscoelastic properties of the pregel solution.

**Figure 7:**
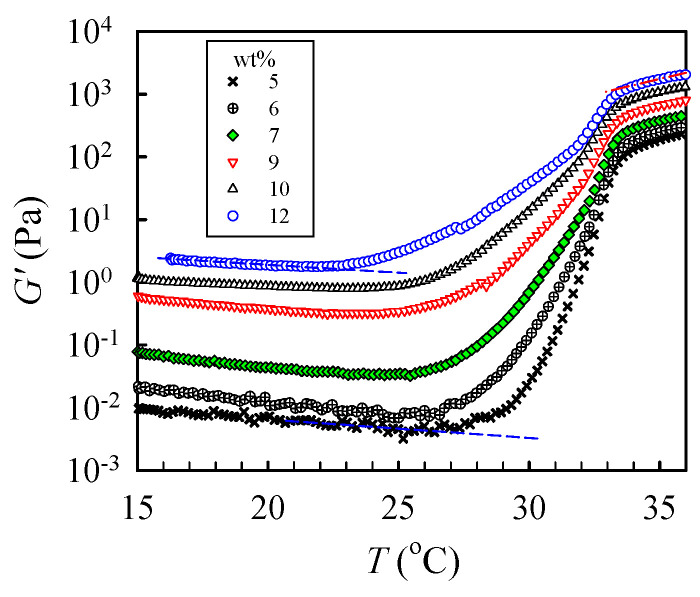
Temperature dependence of the storage modulus *G′* of a-PNIPAM/H_2_O of different concentrations during a temperature sweep test at a heating rate of 0.2 °C/min and applied frequency of 5 rad/s.

To determine the critical temperature needed to reach the pregel regime, a temperature sweep test was performed to detect subtle variations of *G’* at a low heating rate of 0.2 °C /min, as shown in Figure 7. The shapes of the measured *G’* curves were similar for all tested solutions. For the 5 wt% solutions, upon heating from 15 °C, *G′* gradually decreased with increasing temperature until 27.5 °C, at which an upturn was observed. The continuous decrease in *G′* was attributed to an enhanced chain mobility at high temperatures. On the other hand, the upturn of the *G′* curve indicates that the solution structure was altered. The initial temperature at which *G′* upturns is denoted as *T*_1_ to indicate the onset variation of the solution structure and, therefore, the beginning of the pregel regime. Remarkably, a continuous and significant increase in *G′* with a four-order magnitude enhancement at the temperature range of 27–34 °C was observed due to physical gelation and phase separation. With increasing *ϕ*_w_, *T*_1_ was decreased; the derived *T*_1_ was about 22 °C for the 12 wt% solution. Therefore, we conclude that the one-phase solution underwent pronounced concentration fluctuations at temperatures above *T*_1_. The thermal concentration fluctuations subsequently triggered the physical gelation process to develop a macroscopic-scale gel network at *T*_gel_.

**Figure 8:**
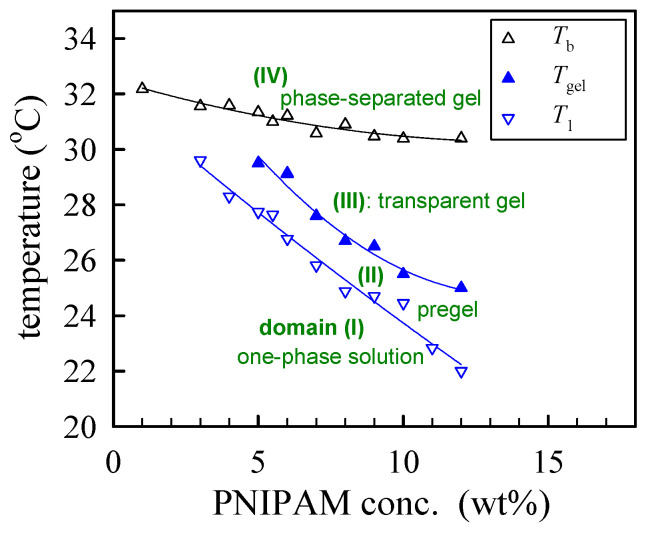
Phase diagram of a-PNIPAM/H_2_O solutions.

The derived phase diagram of the a-PNIPAM aqueous solutions is shown in Figure 8, with *T*_1_ and *T*_gel_ derived from linear viscoelasticity and *T*_b_ derived from turbidity. Both the *T*_gel_ and *T*_b_ were taken from [33]. Depending on the composition and temperature, four domains can be identified in the phase diagram: (I) *T* < *T*_1_, the one-phase solution; (II) *T*_1_ < *T* < *T*_gel_, the pregel solution; (III) *T*_gel_ < *T* < *T*_b_, the transparent gel; and (IV) *T* > *T*_b_, the opaque phase-separated gel.

**Figure 9:**
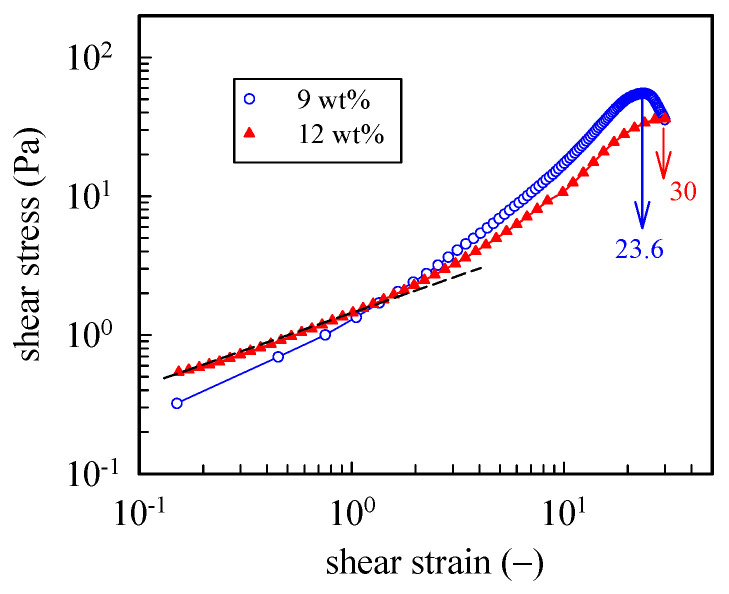
Startup of shear experiment of 9 wt% and 12 wt% a-PNIPAM hydrogels at a temperature 1 °C higher than their *T*_gel_. For both gels, a constant shear rate of 0.05 s^−1^ was applied up to a strain of 30.

To further explore the large strain behavior of the a-PNIPAM hydrogel, we performed startup shear experiments on the 9 wt% and 12 wt% samples at a temperature 1 °C higher than their corresponding *T*_gel_. For both gels, a constant shear rate $\dot{\gamma }$ of 0.05 s^−1^ was applied up to a strain of 30 (*γ*, = $\dot{\gamma }t$). Figure 9 shows the strain dependence of the growing shear stress (*σ*^+^). For the 9 wt% gel, a power law dependence of *σ*^+^ on *γ* was seen in the initial region up to the strain of 100%, at which the linear viscoelastic limit persisted. Further increases in the applied strain led to strain hardening; *σ*^+^ finally reached a peak at *γ* = 2360 and then decreased. Thus, the a-PNIPAM network ruptured at an extremely high strain—a breaking strain much higher than the chemical gels of polydimethylsiloxane (~1000%) [44] and poly (vinyl alcohol) hydrogel (1500%) [45] at their GPs. For the 12 wt% a-PNIPAM networks, similar strain hardening with a higher breaking strain of 3000% was observed. The high breaking strain of an a-PNIPAM network may indicate the existence of a low density of gel junctions with functionality *f* ≥ 3, as shown in our previous work [38]. Moreover, the lifetime of a gel junction must be longer than the time scale of an applied shear rate (~20 s). These results suggest the existence of strong bonding energy in the gel junctions that resist junction breaking before network rupture at high shear strains of 24–30. Thus, a-PNIPAM hydrogel exhibits strong junction strength and demonstrates permanent elasticity at *T*_gel_ < *T* < *T*_b_. This is in great contrast with the transient gel of telechelic PNIPAM [46], which is exclusively involved with van der Waals force within micellar junctions.

It should also be noted that the extremely high elongation at break of the a-PNIPAM hydrogel may support its practical application as a 3D printing and injectable biomaterial [47].

### 2.3. Proposed Mechanism of the Formation of a Gel Junction (Coupled Pearls)

In physical gelation, gel junctions are part of a temporary crosslink via the micro-crystalline region, hydrogen bonding, phase-separated micro-domains, and so on. Considering that a-PNIPAM is a non-crystalline polymer along with the fact that *T*_gel_ < *T*_b_ for the present water solution, the “bonds” that firmly connect the a-PNIPAM chains must mainly be relevant to inter-amide hydrogen bonding.

Based on dielectric relaxation measurements, Ono and Shikata [26] found that the relaxation time for the dehydration process of bound water molecules (i.e., *τ*_ex_ for the process of bound water ⇌ free water) is about 23 ps, which is sufficiently longer than the rotational relaxation of bulk water molecules (i.e., *τ*_w_ for bulk water ⇌ free water) of 8.3 ps. After a residence time of 23 ps, the water molecules hydrated to suitable sites of –CONH– could be readily replaced by free water molecules belonging to the bulk water phase. From a dynamic point of view, the slow exchange process of dehydration may effectively preserve hydration since 1/*τ*_ex_ < 1/*τ*_w_, i.e., free water in correspondence to dehydrated water is readily replenished from the bulk water phase. These results imply that the hydrated NIPAM monomeric units (i.e., the “necklaces” referred to in the pearl-necklace chain model [16]) are safely protected by bound water molecules from exposing their amide groups to the surrounding medium.

**Figure 10:**
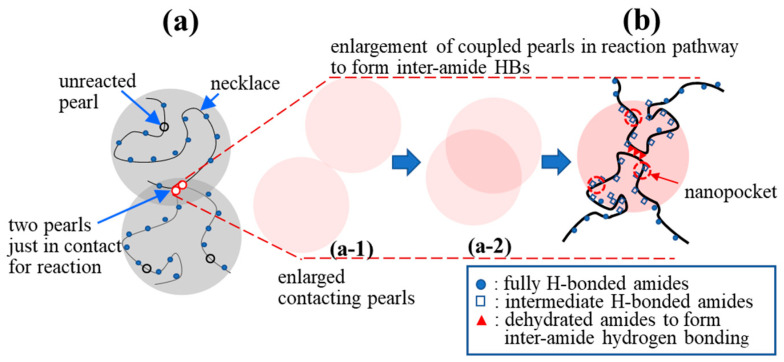
Proposed formation of a physical junction: (**a**) Two a-PNIPAM chains are connected via the hydrophobic interaction of the “pearls” in the overlapping region (red circles, reacted) to form “coupled pearls”; the hydrophilic necklaces contain the fully H-bonded amide groups with 3 bound water molecules, while the hydrophobic pearls contain the dehydrated amide groups with less than 3 bound water molecules; (**b**) enlargement of the “coupled pearls” to illustrate the collapsed chain segments to highlight the interchain association via inter-amide hydrogen bonding. (a-1) and (a-2) show the reaction pathway of the two contacting pearls that gradually overlap and eventually develop the inter-amide HBs in (**b**).

There are no dehydrated NIPAM units in the hydrophilic necklaces used for the cooperative hydration of bound water [16] (Figure 10a). On the one hand, it is difficult for two crossing necklaces to form a gel junction. On the other hand, interchain association is likely to occur between two contacting pearls in which dehydrated segments reside and possess a long enough lifetime to develop coupled pearls via inter-amide hydrogen bond formation. The contacting of the pearls, driven by a hydrophobic interaction, is the first step, followed by “pearl coalescence” that reduces the total hydrophobic surface to eventually develop a “coupled” pearl (Figure 10b). Meanwhile, in the overlapping region of the “coupled pearls”, an inter-amide hydrogen bond (HB) becomes able to develop a firm interchain connection [48]. Figure 10 also illustrates an irreversible physical reaction pathway from part (a-1) to (a-2) and from (a-2) to (b). The irreversible process is stochastic and driven by the gain of free energy brought about by the reduction in enthalpy because of the hydrophobic interaction and inter-amide hydrogen bonding, which outweigh the cost of the free energy caused by the translational and rotational entropy loss of the segments, accompanied by the process from (a-1)→(a-2)→(b).

**Figure 11:**
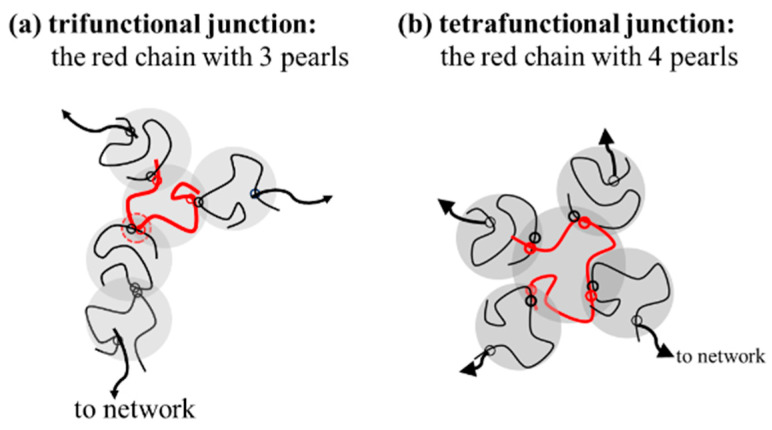
The specific red chains with 3 and 4 pearls acting as (**a**) trifunctional junction (*f* = 3) and (**b**) tetrafunctional junction (*f* = 4).

Based on this proposal, the interpenetration of two pearls of neighboring chains in the overlapping region seems crucial to initiate interchain association, followed by a sequential reaction of inter-amide bonding in the coupled pearls to form a strong bond. It should be noted that a junction with two paths to a gel network, that is, *f* = 2, only extends the length of the network strands. The functionality of the junction should be *f* ≥ 3 for an effective junction to connect the elastically active strands (Figure 11). Thus, a chain with three or more pearls could serve as an effective junction to support external deformation. Through the reaction of the coupling pearls of a-PNIPAM chains in a series, multimers and self-similarly branched chains are developed at elevated temperatures.

### 2.4. Electrospinning of a-PNIPAM/H_2_O Solutions at 10 °C

**Figure 12:**
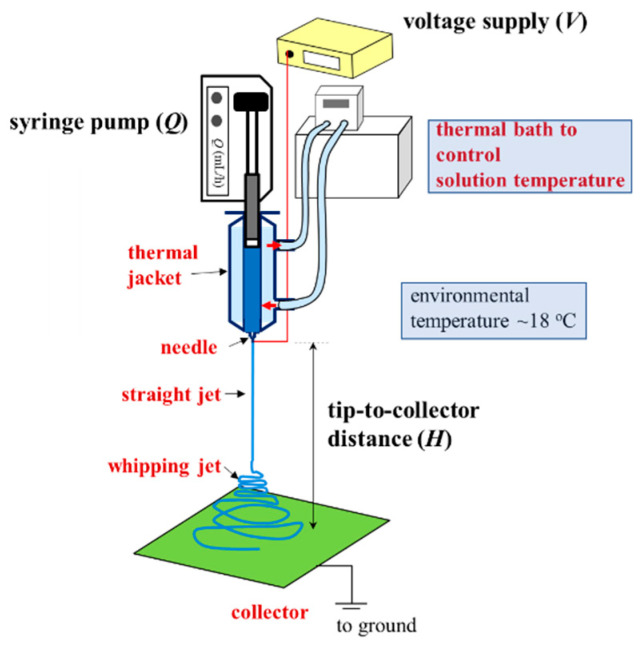
Low-temperature electrospinning process designed to ensure the continuous electrospinning of a one-phase a-PNIPAM aqueous solution. A thermal jacket was used to enclose the polymer solution to maintain a desired temperature (*T*_solution_), controlled by a thermal bath with circulating water. The processing parameters are *Q*, *V* and *H*. The environmental temperature was fixed at 18 °C.

A one-phase polymer solution is preferred for a continuous process of electrospinning to produce uniform nanofibers. Schoolaert et al. [8] obtained uniform PNIPAM fibers by controlling the environmental parameters (e.g., temperature and humidity) without addressing the phase diagram of PNIPAM/H_2_O solutions. Based on the phase diagram (Figure 8), a-PNIPAM/H_2_O solutions are in the one-phase solution regime at 10 °C when *ϕ*_w_ is lower than 15 wt%. On the contrary, at room temperature (around 25 °C), a-PNIPAM solutions with *ϕ*_w_ > 8 wt% may enter the pregel regime; some even develop macroscopic gel when *ϕ*_w_ > 12 wt%. The formation of branched structures and/or physical crosslinks in an electrospinning solution may lead to difficulty in performing the electrospinning process, which produces complicated fiber morphology. To resolve this problem, it is best to maintain an electrospinning solution temperature of lower than *T*_1_. In this work, a thermal jacket was used to maintain the solution temperature (*T*_solution_) by circulating water at a controlled temperature to fulfill this goal. The environmental temperature was also controlled at about 18 °C. The schematics for the low-temperature electrospinning are shown in Figure 12.

#### 2.4.1. Concentration Effect

It has been concluded that the diameter of as-spun fibers decreases with decreasing *ϕ*_w_ [5,6]. However, there is a minimum *ϕ*_w_, below which the entanglement density in a solution is insufficient to support electric stretching forces, thereby yielding non-uniform fibers with subsidiary structures of beads or barbs along them. Another plausible mechanism to produce beaded fibers (or barbed fibers) is associated with “flow-induced phase separation” in the spinline provided that the electric stretching rate is higher than the relaxation rate of a given polymer solution [39]. In any case, it was desirable to find the minimum *ϕ*_w_ of the present a-PNIPAM/H_2_O solution that produced bead-free fibers by controlling the three processing parameters (applied voltage *V*, flow rate *Q,* and tip-to-collector distance *H*).

**Figure 13:**
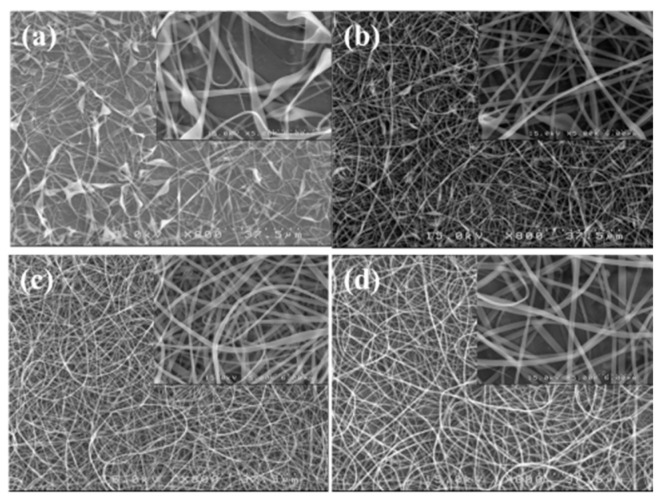
SEM images of fibers electrospun from a-PNIPAM aqueous solutions at different concentrations of (**a**) 8, (**b**) 9, (**c**) 10, and (**d**) 14 wt%. (*Q* = 0.1 mL/h, *V* = 13 kV, *H* = 21 cm, *T*_solution_ = 10 ºC.)

A processing window (*V* vs. *Q*) at a constant *H* of 21 cm was initially constructed to determine the common processing parameters used to subsequently electrospin a-PNIPAM solutions with different *ϕ*_w_. Based on the processing window, the following parameters were applicable to all the solutions (*ϕ*_w_ = 7–14 wt%) studied to achieve a stable “cone-jet” electrospinning mode: *Q* = 0.1 mL/h, *V* = 13 kV, and *H* = 21 cm. When using these processing parameters, the electrospinning process was stable for more than 1 h. SEM images of the collected fibers are shown in Figure 13; the inset shows a higher magnification. Fiber structures with symmetric beads (spindles) and asymmetric barbs were still observed at *ϕ*_w_ = 8 wt% (= *ϕ*_e_). The number of spindle and barbs and their sizes were decreased for the a-PNIPAM fibers electrospun from the 9 wt% solutions. To obtain bead-free a-PNIPAM fibers, a minimum concentration of 10 wt% was required, thus giving rise to an *ϕ*_w_/*ϕ*_e_ value of 1.4. This finding is consistent with the previous suggestion that sufficient chain entanglements in a given solution are generally required to produce uniform fibers [5]. Meanwhile, for the a-PNIPAM electrospun from the 14 wt% solutions, the as-spun a-PNIPAM fibers possessed an average fiber diameter of 600 ± 180 nm, based a collection of 200 fibers. Thus, by carefully controlling the temperature and polymer concentration for electrospinning, round a-PNIPAM fibers with a diameter of 450–600 nm were successfully derived from the entangled solutions.

It is known that beaded fibers and/or barbed fibers are obtained as ϕ* < *ϕ*_w_ < *ϕ*_e_, where ϕ* is the overlap concentration, at which the random coils of neighboring polymer chains start to overlap one another. As *ϕ*_w_ < ϕ*, particulates are primarily produced on the grounded collector; in other words, the electrospinning is degenerated and becomes electrospraying [6,41].

#### 2.4.2. Flow Rate Effect

**Figure 14:**
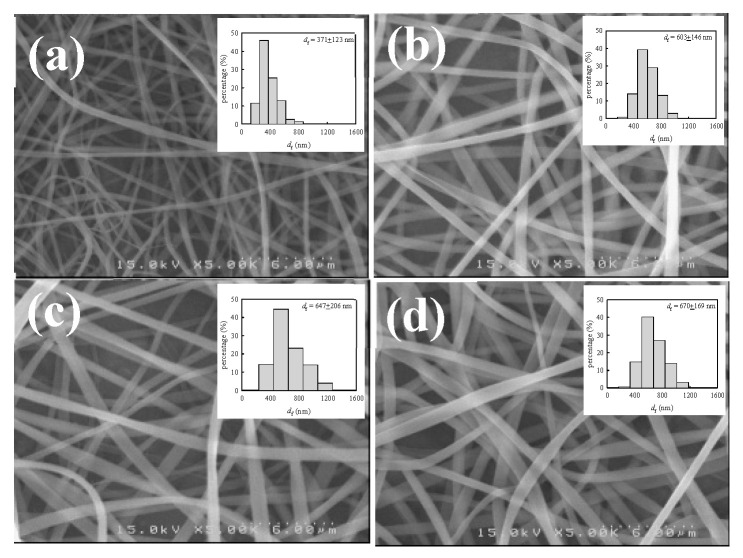
SEM images and diameter distributions of a-PNIPAM fibers electrospun at a given flow-rate of (**a**) 0.05, (**b**) 0.1, (**c**) 0.15 and (**d**) 0.2 mL/h. (12 wt% solution, *V*= 17 kV, *H*= 21 cm, *T*_solution_= 10 ºC.).

For a given solution of *ϕ*_w_, the diameter of as-spun fibers (*d*_f_) depends on the interplay of the processing parameters (*V*, *Q*, and *H*). There are several scaling laws used to describe the relation between *d*_f_ and the processing parameters [6]. In general, *d*_f_ is decreased with decreasing *Q* and an enhanced electric field strength of *V*/*H* (thereby increasing *V* and/or decreasing *H*). Among these parameters, *Q* is the most important in determining the fiber diameter for a given polymer solution [41]. To explore the *Q* effect, the 12 wt% a-PNIPAM solution at 10 °C was used for electrospinning at different *Q*, ranging from 0.05 mL/h to 0.2 mL/h, by fixing *V* = 17 kV and *H* = 21 cm. The fiber morphology of the as-spun fibers is shown in Figure 14, together with a histogram of the measured fiber diameters. It should be noted that bead-free fibers were obtained. The average fiber diameters electrospun from *Q* = 0.05, 0.1, 0.15, and 0.2 mL/h were 371 ± 123, 603 ± 146, 647 ± 206, and 670 ± 169 nm, respectively. The results show that *d*_f_ was approximately doubled, with a four-fold increase in *Q* from 0.05 mL/h to 0.2 mL/h.

### 2.5. Electrospinning of a-PNIPAM/H_2_O Solution at Different Temperatures

**Figure 15:**
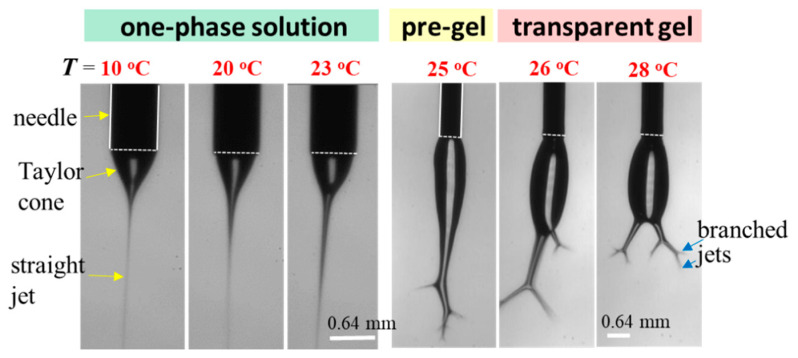
Effect of solution temperature on the cone/jet morphology during the electrospinning of the 10 wt% a-PNIPAM/water mixture. The scale bar is 0.64 mm. The bright line appearing on the cone was caused by the light source used for illumination.

**Figure 16:**
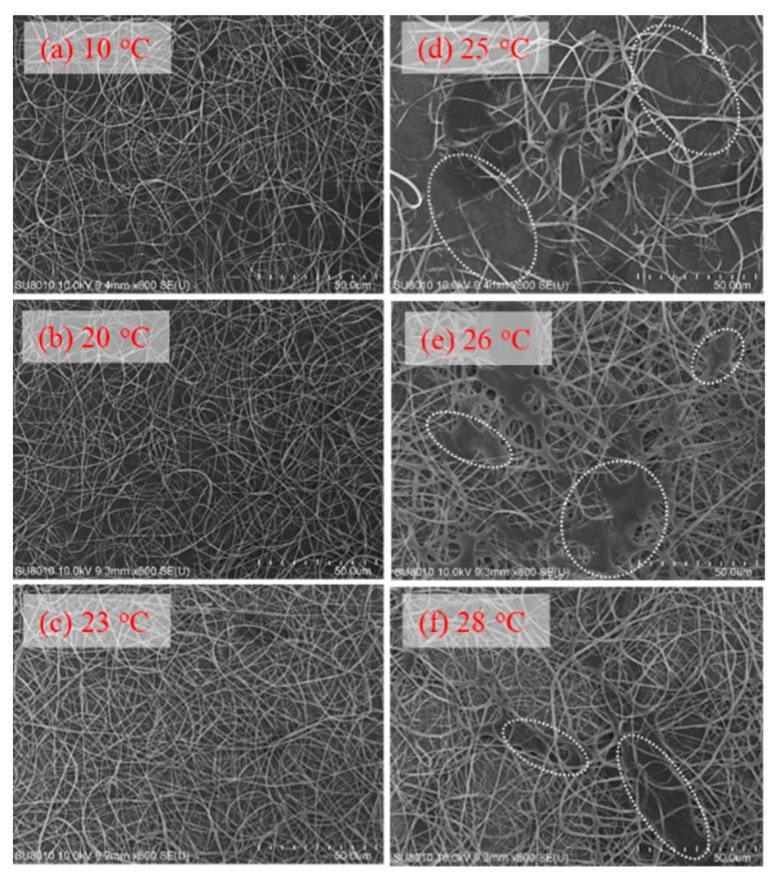
Effect of solution temperature on the fiber morphology collected on the grounded collector (**a**–**f**). In (**d**–**f**), the white dotted regions indicate the area where the detaching droplets splashed onto the nanofiber fabrics.

To explore the effect of solution temperature (*T*_solution_), the 10 wt% a-PNIPAM solution was used for electrospinning by holding *T*_solution_ at constant temperatures of 10 °C, 20 °C, 23 °C, 25 °C, 26 °C, and 28 °C. Based on Figure 8, these selected *T*_solution_s covered the phase domains of (I) one-phase solution (e.g., 10 °C, 20 °C, and 23 °C), (II) pregel (e.g., 25 °C), and (III) transparent gel (e.g., 26 °C and 28 °C) given that *T*_1_ and *T*_gel_ were 24.5 °C and 25.5 °C, respectively. Figure 15 shows the morphologies of the Taylor cone and straight jet during electrospinning, and SEM images of collected a-PNIPAM fibers are displayed in Figure 16 for comparison. For the 10 wt% solutions in the one-phase state, the stable cone-jet electrospinning mode could be maintained to continuously produce nanofibers. Dried a-PNIPAM nanofibers were obtained on the grounded collector. As *T*_solution_ was increased from 10 °C to 20 °C and 23 °C, the measured *d*_f_ values were 570 ± 150, 580 ± 160, and 700 ± 190 nm, respectively. The abrupt increase in fiber diameter at *T*_solution_ = 23 °C was unexpected because the solution viscosity decreased with increasing temperature before reaching the pregel domain (*T* > *T*_1_). However, this may suggest that flow-induced phase separation also plays a role in the process, as the interchain associations were slightly enhanced to dramatically reduce the relaxation rate of the solution with branched-chain structures.

An unstable electrospinning mode was seen as at *T*_solution_ ≥ 25 °C, at which the 10 wt% a-PNIPAM/H_2_O mixture was either in the pregel state or in the transparent gel state. In these states, gel elasticity played a dominant role during electrospinning in resisting electrical stretching. Figure 15 shows that the viscoelastic droplet, adhering to the needle end (dashed line), was elongated enough to eject one main jet (or two) at its bottom section that subsequently ejected branched sub-jets. It is intriguing to notice that jet whipping was not seen at the end of the branched jet. Instead, the internal structures in the branched jet were directly spat out, eventually producing nanofibers on the grounded collector. In other words, jet whipping is not a prerequisite process to produce the as-spun fibers. As the electrospinning was continuous, the elongated droplet became longer, and some sections of the jet became thinner; eventually, the thinnest section broke. On the one hand, as the jet was broken, the pending droplets and remaining jet were reduced in size without interrupting the electrospinning process; hence, dried a-PNIPAM fibers were continuously produced on the grounded collector. On the other hand, the broken part of the charged jet driven by electric forces under the electric field quickly flew to reach the collector. Considering that solvent evaporation was very limited, the broken jet was still wet after arriving at the collector. Therefore, splashing was readily seen on the grounded collector, along with dried fibers. Some previously deposited dried fibers may have been dissolved because of the splashing of the wet jets, as shown by the dotted regions in Figure 16. These results are in contrast with those in the stable cone-jet mode, which only produced dried fibers on the grounded collector.

**Figure 17:**
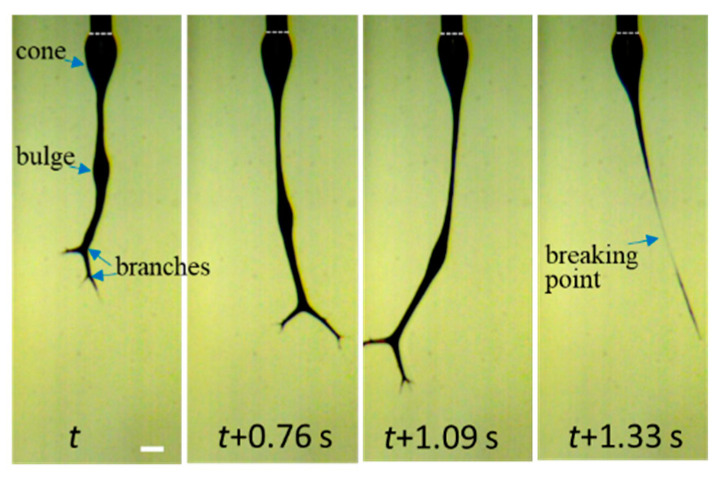
Four snapshots recorded by high-speed video to elucidate the details of jet breaking for the 10 wt% a-PNIPAM aqueous solution electrospun at *T*_solution_ = 28 °C. The scale bar is 0.64 mm.

To enable the better observation of the jet-breaking event, a high-speed video with a frame rate of 3000 fps is provided in Supplementary Materials Video S1, from which four snapshots are shown in Figure 17 to reveal details. The imbalanced electric forces, induced by the surface charges and the electric field, were significantly increased and vigorously vibrated the “droplet-jet”, thus leading to the jet rupture.

We conclude that the continuous electrospinning of transparent a-PNIPAM gel at 28 °C is feasible, despite the fact that a steady state of “cone-jet” morphology is never possible and the production of by-products is inevitable. The feasibility of electrospinning an a-PNIPAM gel is attributed to the gel’s low elastic modulus (*G*_e_ ~13 Pa at 29 °C; see Figure 4d), which means that its crosslinking density is extremely low. For the ultra-soft gel of an a-PNIPAM hydrogel to be processed, the as-spun fibers must possess an average diameter of about 740 ± 330 nm. Accordingly, the a-PNIPAM fibers electrospun from the hydrogel at 28 °C possessed a larger *d*_f_ and a wider fiber distribution compared to that obtained from a one-phase solution at 10–23 °C. The enhanced elasticity of an electrospinning solution resists the stretching stress induced by an electric field, thereby yielding thicker fibers. Furthermore, continuous electrospinning becomes problematic if an a-PNIPAM hydrogel is in the phase-separated state (domain IV, Figure 8), likely because gel elasticity is so high that it dramatically resists stretching electrical forces.

## 3. Conclusions

Understanding the rheological properties of an electrospinning solution and its related phase diagram is necessary to realize the better morphology control of as-spun fibers. In a temperature range of 25–31 °C, semi-diluted a-PNIPAM aqueous solutions were found to exhibit a sol–gel transition and LCST-type phase separation. Moreover, even in a thermodynamically stable solution at a temperature slightly lower than *T*_1_, the solution possibly underwent flow-induced concentration fluctuation and phase separation. Hence, these phase transitions, if occurring in the spinline, will interfere with or even interrupt a solution’s processibility because of the formation of a complex fluid with physical gels and/or a phase-separated structure. Therefore, selecting a suitable processing temperature for solution electrospinning is the most important issue to address in the process. In this study, we constructed the phase diagram of an a-PNIPAM aqueous solution that can serve as a guideline for electrospinning. When electrospun at temperatures lower than *T*_1_, entangled solutions were found to produce essentially uniform a-PNIPAM nanofibers with a diameter of 450–600 nm.

In addition, the a-PNIPAM hydrogel was found to possess a low elasticity of 10–20 Pa and an extremely high elongation at break (2400–3000%) at ambient temperature. These properties facilitate its potential application as a 3D printing and injectable biomaterial.

## 4. Materials and Methods

### 4.1. Solution Preparation and Properties

a-PNIPAM was obtained from Scientific Polymer Products Inc. (Ontario, NY, USA). The *meso* diad content was determined to be 48% from a ^1^H-NMR spectrum by using an ECZ-400S spectrometer (JEOL Ltd., Tokyo, Japan) with DMSO-*d*_6_ as a solvent at 423 K. The weight-average molecular weight and polydispersity were determined to be 6.58 × 10^5^ g/mol and 1.49, respectively [38]. De-ionized water was used as a solvent to prepare the electrospinning solutions. Different amounts of polymers and solvents were vigorously mixed at 10 °C for three days to prepare the one-phase solutions of different concentrations, followed by storage in a freezer at 5 °C prior to measurements. Cloud points were derived from turbidity under an optical microscope [38]. Upon heating from 10 °C at a fixed rate of 0.05, 0.2, 0.5 and 1.0 °C/min, transmitted light intensity was measured as a function of temperature. For a given heating rate, the cloud point temperature (*T*_cp_) was derived from the onset temperature of turbidity.

The linear viscoelastic properties of the a-PNIPAM solutions were measured with a rheometer (ARES) by using a cup-and-bob fixture under a small-amplitude oscillatory shear mode [38]. To reveal the phase transition, an isothermal frequency sweep test was performed to obtain the frequency (*ω*) dependence of the storage modulus *G′(ω)* and loss modulus *G″(ω)*. During the frequency sweep, the appropriate strain amplitude was applied according to the preliminary strain sweep test to ensure the conditions of linear viscoelasticity and sufficient torque for data collection. The complex viscosity η* was then calculated using the equation $[G^{\prime }{\left(\omega \right)}^{2}+G^{\prime \prime }{\left(\omega \right)}^{2}{]}^{0.5}/\omega$.

### 4.2. Electrospinning and Fiber Morphology

A thermal jacket was used to enclose the electrospinning solution to control the solution temperature (*Ts*) by using circulating water at different temperatures. Electrospinning was performed in a specific room with an environment temperature of about 18 °C. The polymer solution with a determined temperature (10–30 °C) was delivered by a syringe pump (Cole–Parmer, Vernon Hills, IL, USA) at a controlled flow rate of (*Q*) through PTFE tubing into the stainless needles (Hamilton, outer diameter = 0.64 mm). A high electrical voltage (Bertan, 205B, USA) was applied to the needles. To construct a needle-plate electrode configuration, we used a steel net (30 × 30 cm^2^) to collect electrospun fibers at a tip-to-collector distance of 21 cm below the needle tip. The morphologies of the Taylor cone and electrospinning jet were monitored by using a high-speed video system. The morphology and diameter of as-spun fibers were observed and measured with a scanning electron microscope (SEM, Hitachi S4100, Tokyo, Japan).

## Supplementary Materials

The following supporting information can be downloaded at: https://www.mdpi.com/article/10.3390/gels8110716/s1, Video S1: high-speed video of the electrospinning jet.
